# Investigating links between white matter hyperintensities and menopausal status using robust age-correction methods in UK Biobank

**DOI:** 10.1093/braincomms/fcaf475

**Published:** 2025-12-05

**Authors:** Denise Wezel, Olivier Parent, Manuela Costantino, Lina Sifi, Grace Pigeau, Nicole J Gervais, Ann McQuarrie, Josefina Maranzano, Gabriel A Devenyi, Mahsa Dadar, M Mallar Chakravarty

**Affiliations:** Cerebral Imaging Center, Douglas Mental Health University Institute, H4H 1R3 Montreal, QC, Canada; Faculty of Behavioural and Movement Sciences, Vrije Universiteit Amsterdam, 1081 HV Amsterdam, the Netherlands; Cerebral Imaging Center, Douglas Mental Health University Institute, H4H 1R3 Montreal, QC, Canada; Integrated Program in Neuroscience, McGill University, H3A 1A1 Montreal, QC, Canada; Department of Genetic Medicine, University of Chicago, 60637 Chicago, IL, USA; Cerebral Imaging Center, Douglas Mental Health University Institute, H4H 1R3 Montreal, QC, Canada; Integrated Program in Neuroscience, McGill University, H3A 1A1 Montreal, QC, Canada; Cerebral Imaging Center, Douglas Mental Health University Institute, H4H 1R3 Montreal, QC, Canada; Integrated Program in Neuroscience, McGill University, H3A 1A1 Montreal, QC, Canada; Groningen Institute for Evolutionary Life Sciences, Faculty of Science and Engineering, Rijksuniversiteit Groningen, 9712 CP Groningen, The Netherlands; Faculty of Anatomy and Cell Biology, McGill University, H3A 0C7 Montreal, QC, Canada; Faculty of Medicine, McGill University, H3G 2M1 Montreal, QC, Canada; Department of Anatomy, Université du Québec à Trois-Rivières, G8Z 4M3 Trois-Rivières, QC, Canada; Cerebral Imaging Center, Douglas Mental Health University Institute, H4H 1R3 Montreal, QC, Canada; Department of Psychiatry, McGill University, H3A 1A1 Montreal, QC, Canada; Cerebral Imaging Center, Douglas Mental Health University Institute, H4H 1R3 Montreal, QC, Canada; Department of Psychiatry, McGill University, H3A 1A1 Montreal, QC, Canada; Cerebral Imaging Center, Douglas Mental Health University Institute, H4H 1R3 Montreal, QC, Canada; Integrated Program in Neuroscience, McGill University, H3A 1A1 Montreal, QC, Canada; Department of Psychiatry, McGill University, H3A 1A1 Montreal, QC, Canada; Department of Biomedical Engineering, McGill University, H3A 2B4 Montreal, QC, Canada

**Keywords:** white matter hyperintensities, menopause, cardiometabolic risk factors, brain aging

## Abstract

White matter hyperintensities (WMHs) are radiological abnormalities indicative of cerebrovascular dysfunction associated with increased risk for cognitive decline. WMHs increase in prevalence with older age, and there are known sex differences as older women harbour higher WMH burden than men. Some have hypothesized that the increase in this dementia-related risk factor is related to the menopausal transition.

To untangle the effects of age and menopause, we leveraged a large cross-sectional sample of women from the UK Biobank (*n* = 9560) to investigate differences in WMH volumes across menopausal status using a strict age-matching procedure.

Surprisingly, we find higher WMH volumes in premenopausal women compared to postmenopausal women in certain analysis schemes, especially as compared to surgically postmenopausal women. These results reached significance mostly in analyses where the postmenopausal groups had a longer time since menopause. Our results pertaining to menopause-related characteristics, such as age at menopause or menopause hormonal therapy, did not replicate the literature reporting an association with WMH volumes. Cardiometabolic factors, such as smoking and blood pressure, were significant predictors of WMH volume in the full sample without age-matching. These effects were not significantly different across menopausal status, with the exception of blood pressure medication use, which was associated with higher WMH volumes to a larger extent in premenopausal women relative to postmenopausal women.

Our findings are in the opposite direction of reported effects of higher WMH volumes following the menopausal transition, which could be due to variations in age correction techniques or idiosyncrasies in the UK Biobank sample, especially as it relates to the lack of data on perimenopause. We further show that the effects of positive cardiometabolic and lifestyle factors on brain health, as indexed with WMH volumes, generally do not change after menopause. Factors other than the menopausal status may be at play in explaining the difference in WMH burden between men and women in later life.

## Teaser

Wezel, Parent *et al*. investigated white matter hyperintensity volumes across the menopausal transition in the UK Biobank using strict age-matching procedures. Postmenopausal women did not exhibit elevated white matter hyperintensity volumes; in fact, volumes were lower under certain analytic schemes, suggesting potential idiosyncrasies of the UK Biobank dataset.

## Graphical Abstract

**Graphical Abstract:**
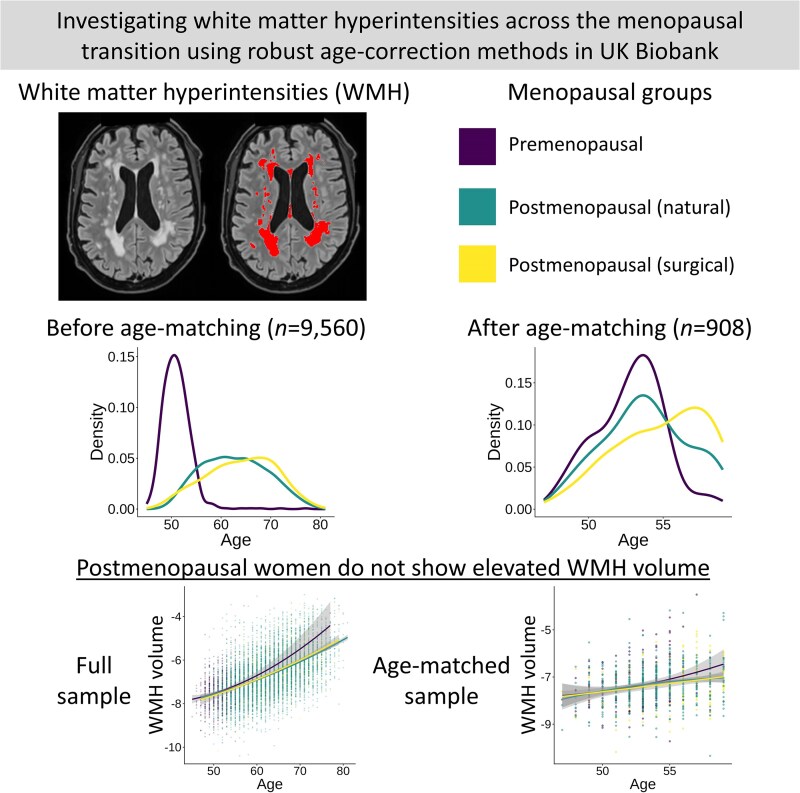

## Introduction

White matter hyperintensities (WMHs) are radiological abnormalities that appear on T2-weighted MRI scans^1^ and are recognized markers of small vessel disease and cerebrovascular dysfunction.^2^ WMH burden is strongly predicted by age^3^ and is associated with maladaptive aging,^4^ increased risk for neurovascular pathologies such as stroke,^1^ cognitive decline and dementia,^5^ decreased gait speed,^6^ and overall mortality.^1^ Taken together, WMH burden represents a critical neuropathological marker across the neurodegenerative spectrum.

Previous reports show evidence of sex differences in WMH burden, with elderly women demonstrating a higher WMH burden relative to age-matched males,^7-9^ although hospital-based samples sometimes show higher WMH burden in males.^10^ Recent work from our group has uncovered nuanced sex differences, with women having higher WMH volumes in periventricular and anterior regions and men having higher WMH volumes in posterior regions.^11^ However, these sex differences are not observed in younger samples,^9,12^ suggesting their emergence later in life. The neuroprotective effects of circulating oestrogen^13,14^ may explain this difference, suggesting that the menopausal transition influences women’s WMH burden.^15^

The decrease in ovarian hormones due to natural menopause (typically occurring between ages 45 and 55) influences various physiological systems, including brain structure and function, although findings remain inconsistent.^16-18^ The menopausal transition is associated with an increase in cardiovascular risk factors, such as hypertension and diabetes,^19,20^ that double as risk factors for WMHs.^21-23^

Longer periods of ovarian hormone cessation due to earlier menopause onset are associated with WMHs.^23,24^ Further, postmenopausal women are reported to have higher WMH burden than premenopausal women,^8,25^ a finding supported by increases in WMHs observed in recently menopausal women.^26^ Consistent with the oestrogen neuroprotection hypothesis, surgical menopause (where oophorectomy occurs before the onset of natural menopause^27^) has been associated with an increased risk of cognitive impairment, dementia,^28^ and neurodegeneration.^29,30^ However, results are mixed. Previous findings from our group revealed no menopause-related changes in brain volume following a strict age-matching procedure in a large sample.^31^ Zeydan *et al*. (2019) report no difference in WMH burden in surgical compared to natural menopause.^29^ Studies of the impact of menopause hormonal therapy (MHT; also known as hormone replacement therapy) during the menopause transition, often considered to be neuroprotective, remain inconclusive: some studies report mixed conclusions,^32,33^ while others report no neuroprotective impact of MHT.^23,34^

Historically, many previous studies examining the relationship between menopause and brain health can be generally categorized into two types: (i) small-sample studies that recruit age-matched controls (usually males) (e.g.^29,35^) or that do not age-match;^24,26^ (ii) larger cohort studies that do not age-match.^8,25^ Without age-matching, the difference in average age between premenopausal and postmenopausal women can be as much as^14^ years.^25^ In some studies, the mean age per group was unreported.^8^ In both designs, disentangling the neurobiological impact of the menopause transition independent of sex-specific age-related neurodegeneration is challenging. On the other hand, large population-based studies may find it difficult to recruit age-matched controls.

Here, we analysed a large sample from the UK BioBank and performed the age-matching procedure previously championed by our group^31^ to disentangle the effects of age and both surgical and natural menopause on white matter hyperintensity volume (WMHV). We also performed additional analyses to replicate findings in the study by Lohner and colleagues (2022), who reported significant effects of menopausal status on WMHV in the large population-based Rhineland Study.^8^ We further analysed whether WMH burden is influenced by age at self-reported menopause and MHT use, as well as the interplay between cardiometabolic factors and WMH volumes before and after menopause.

## Materials and methods

### Sample

The present study uses cross-sectional data obtained from the UK Biobank^36^ under application number 45551. The study was approved by the North West Multicenter Research Ethics Committee, and all participants provided their written informed consent. The UK Biobank is a representative population-based study with 39 676 participants from the United Kingdom who underwent brain MRI scans between 2016 and 2022. We categorized participants into three groups based on self-reported data: premenopausal women (PRE), women who underwent natural menopause (POST) and women who underwent surgical menopause (SURG; bilateral oophorectomy before onset of natural menopause, with or without a hysterectomy). Time since menopause was calculated by subtracting age at menopause from age at the time of scanning. Lifetime use of MHT was encoded as a binary variable (yes/no). Diagnoses of stroke, multiple sclerosis and diabetes were taken from the first occurrences data field (category #1712), where data from self-reported medical conditions, primary care, hospital inpatient and death register records were mapped to ICD-10 codes together with an estimated date of diagnosis.

A flowchart of exclusions is available in Supplementary Fig. 1. Participants were excluded from the study if they had missing MRI data (*n* = 1006) or motion artifacts during the T1-weighted scan (*n* = 6970), if they had a diagnosis of stroke before the MRI visit or a lifetime diagnosis of multiple sclerosis (*n* = 118), if they underwent a hysterectomy without a oophorectomy (*n* = 578), if they had missing menopausal group data (*n* = 695) and if they had missing data on income or MHT use (*n* = 1391). We further excluded postmenopausal women with missing data on age at menopause (*n* = 610), premenopausal women reporting MHT use (*n* = 58) and women with a male genetic sex (*n* = 4). The full sample included in the present study consists of 9560 women between 45 and 81 years old and is similar to the sample used in our previous work by Costantino *et al*. (2023).^31^ In each analysis, different subsamples of the groups were included based on data availability and age-matching. Table 1 shows an overview of descriptive statistics for each subgroup. Table 2 clarifies which dataset is used in which analysis.

**Table 1:** Demographics of participants per group

	(A) Full sample (unmatched) (*n* = 9560)		
	PRE (*n* = 674)	POST (*n* = 7992)	SURG (*n* = 894)
Age	51.04 (2.89; 45–77)	62.73 (6.58; 47–81)	63.91 (6.92; 47–79)
Age at menopause	N/A	50.72 (4.76)	46.49 (6.73)
Years since menopause	N/A	12.00 (7.74)	17.41 (8.74)
Income a	46/103/190/250/85	1053/2320/2414/1705/500	132/284/277/155/46
MHT users	0 (0%)	2604 (33%)	713 (80%)

	(B) Nearest-neighbour age-matched sample (*n* = 908)		
	PRE (*n* = 223)	POST (*n* = 454)	SURG (*n* = 231)
Age	52.75 (2.30; 47–59)	53.81 (2.88; 47–59)	54.84 (3.01; 47–59)
Age at menopause	N/A	49.44 (3.92)	44.95 (6.01)
Years since menopause	N/A	4.37 (4.03)	9.89 (6.36)
Income a	11/36/78/68/30	24/92/119/160/59	28/44/75/61/23
MHT users	0 (0%)	109 (24%)	157 (68%)

	(C) Exact age-matched sample (*n* = 536)		
	PRE (*n* = 134)	POST (*n* = 268)	SURG (*n* = 134)
Age	52.93 (2.46; 47–59)	52.93 (2.46; 47–59)	52.93 (2.46; 47–59)
Age at menopause	N/A	49.30 (3.78)	44.60 (6.00)
Years since menopause	N/A	3.63 (3.73)	8.34 (6.14)
Income a	8/21/51/43/11	13/50/73/90/42	17/21/44/37/15
MHT users	0 (0%)	67 (25%)	94 (70%)

	(D) POST–SURG age-matched sample (*n* = 1788)	
	(D^x^) POST (*n* = 894)	(D^y^) SURG (*n* = 894)
Age	63.91 (6.92; 47–79)	63.91 (6.92; 47–79)
Age at menopause	50.57 (4.82)	46.50 (6.73)
Years since menopause	13.34 (7.95)	17.41 (8.74)
Income a	121/268/283/177/45	132/284/277/155/46
MHT users	306 (34%)	713 (80%)

	(E) Lohner *et al*. (2022) replication sample (*n* = 3669)	
	PRE (*n* = 668)	POST (*n* = 3001)
Age	50.89 (2.36; 45–59)	55.50 (2.67; 45–59)
Age at menopause	N/A	49.68 (4.47)
Years since menopause	N/A	5.82 (4.66)
Income a	45/100/188/250/85	263/591/879/934/334
MHT users	0 (0%)	778 (26%)

	(F) > 5 years after menopause age-matched sample (*n* = 526)		
	PRE (*n* = 126)	POST (*n* = 263)	SURG (*n* = 137)
Age	53.53 (2.22; 48–59)	54.38 (2.48; 48–59)	55.15 (2.46; 48–59)
Age at menopause	N/A	45.44 (4.31)	42.42 (5.41)
Years since menopause	N/A	8.94 (3.38)	12.73 (5.13)
Income a	6/22/51/32/15	27/45/80/85/26	15/27/45/36/14
MHT users	0 (0%)	95 (36%)	103 (75%)

For age, age at menopause and time since menopause, the mean and standard deviation (in parentheses) are shown. The number of participants who used MHT, as well as the percentage of the sample, is also reported.

MHT = Menopause hormonal therapy.

^a^For income, the values indicate the number of participants in each of the following total household income categories (in pounds sterling): <18 000/18 000–30 999/31 000–51 999/52 000–100 000/>100 000.

**Table 2:** Overview of all models and datasets used

Differences in WMHV across menopausal status	
Model	Sample(s)
WMHV ∼ group + age + income + MHT + (1|MRI site)	A
WMHV ∼ group + poly(age,2) + income + MHT + (1|MRI site)	A, B, C, F
WMHV ∼ group + poly(age, 2) + income + MHT + (1|MRI site) + cardiometabolic factors	A, B, C, F
WMHV ∼ group + age + income + (1|MRI site) + hypertension + smoking status + past tobacco + pack years + BMI	E

Effect of age at menopause and MHT use on WMHV	
Model	Sample(s)
WMHV ∼ age at menopause + poly(age, 2) + income + MHT + (1|MRI site)	D^x^, D^y^
WMHV ∼ group × age at menopause + poly(age, 2) + income + MHT + (1|MRI site)	D
WMHV ∼ MHT + group + poly(age, 2) + income + age at menopause + (1|MRI site)	D,
WMHV ∼ MHT + poly(age, 2) + income + age at menopause + (1|MRI site)	D^x^, D^y^
WMHV ∼ MHT × group + poly(age, 2) + income + (1|MRI site)	D
WMHV ∼ MHT × age at menopause + poly(age, 2) + income + (1|MRI site)	D^x^, D^y^

Effect of menopausal status on the relationships between cardiometabolic factors and WMHV	
Model	Sample(s)
WMHV ∼ cardiometabolic variable + poly(age, 2) + income + MHT + (1|MRI site)	A
WMHV ∼ cardiometabolic variable × group + poly(age, 2) + income + MHT + (1|MRI site)	B
WMHV ∼ group × BP medication + diastolic blood pressure + systolic blood pressure + poly(age, 2)+ income + (1|MRI site)	B

Table shows all models that were run, in order of mention in the results section. Models were run using different datasets, which is specified by the letter in the sample(s) column (A: full unmatched sample; B: nearest-neighbour age-matched sample; C: exact age-matched sample; D: POST–SURG age-matched sample; E: Lohner *et. al*. 2022 replication sample; F: >5 years after menopause age-matched sample).

### MRI data

**Figure 1:**
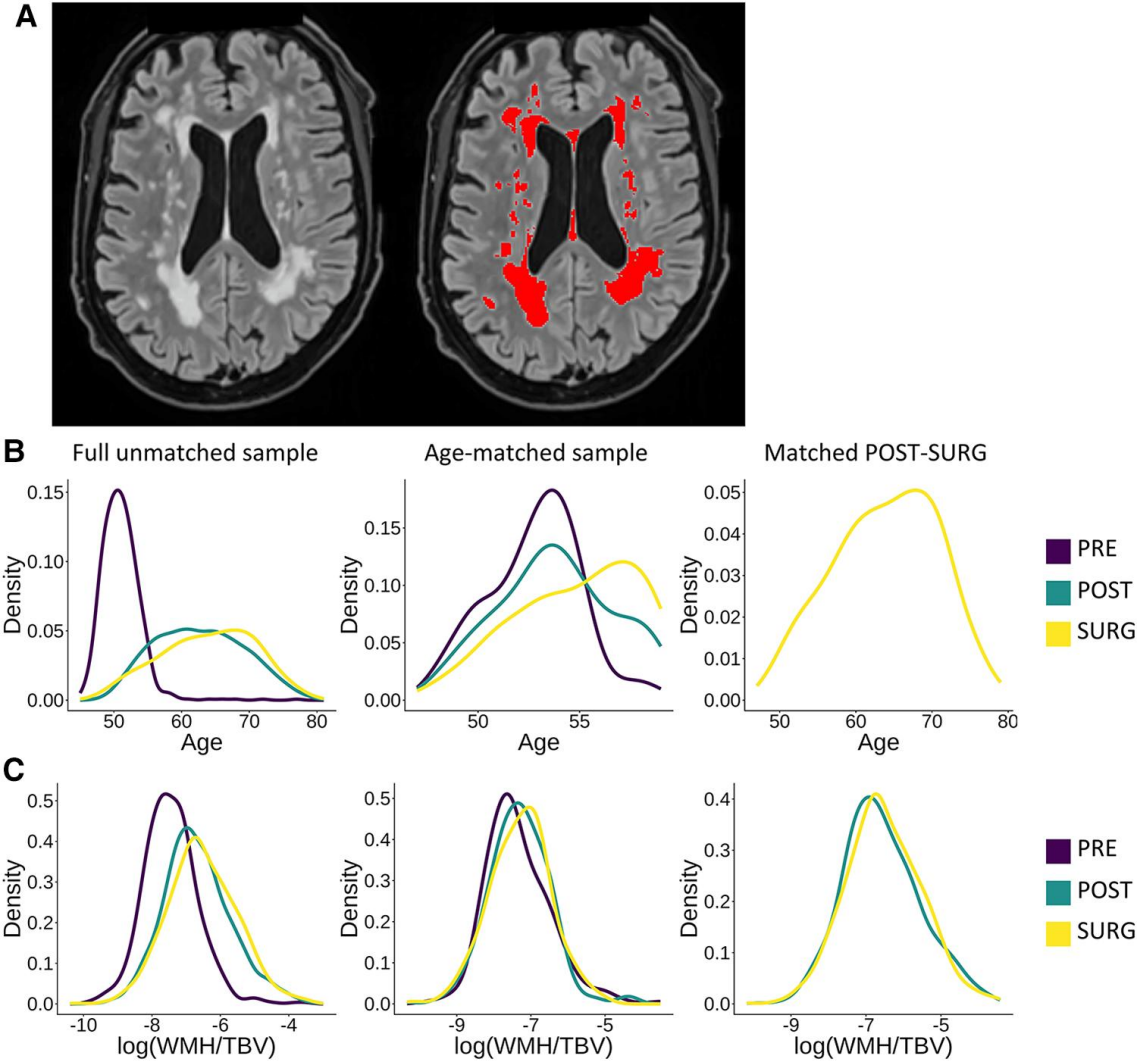
**WMH segmentation and age-matching procedure. (A)** An example of WMH segmentation from the UK Biobank. The left image shows the preprocessed FLAIR image, the right image shows the BISON WMH mask. (**B)** Age distributions before and after age-matching. The middle panel shows the nearest-neighbour age-matched sample. (**C)** WMHV distributions before and after age-matching. WMH: White matter hyperintensities. TBV: Total brain volume. PRE: Premenopausal women. POST: Women who underwent natural menopause. Surg: Women who underwent surgical menopause.

All MRI data were collected from three centers in the United Kingdom using a standardized protocol (Siemens Skyra 3T, with a 32-channel RF receive head coil).^37^ While three different sites were used to acquire MRI data, all scanning parameters and scanner hardware are harmonized across sites. T1-weighted (T1w), fluid-attenuated inversion recovery (FLAIR) and diffusion-weighted imaging acquisitions were used (acquisition parameters are detailed in Supplementary Methods 1). Image processing steps were undertaken as part of a broader effort to reprocess UK Biobank MRI data to maximize registration and segmentation accuracy, detailed in Supplementary Methods 2 and in Parent *et al*. 2025.^11^ Sensitivity analyses were performed with UK Biobank-derived WMHV values. Briefly, to increase the accuracy of between-subject spatial alignment in the white matter, both T1w and fractional anisotropy maps were used to generate a representative population-based template,^38^ to which individual participants were aligned using non-linear registration.^39^ Next, we retrained the BraIn SegmentatiON (BISON) algorithm using manually labelled WMHs of 60 UK Biobank participants,^40^ and then used this trained algorithm to segment WMHs using both T1w and FLAIR as inputs. Of note, for this manuscript, the registration was only used to transform priors into native space, informing tissue prevalence for the BISON algorithm. BISON WMH segmentations were highly correlated with UK Biobank-derived values computed with BIANCA from FSL (*r* = 0.86, *P* < 0.001), and in cases of discrepancy, visual assessment favoured BISON segmentations. This procedure thus ensured a high segmentation accuracy of WMHs. A WMH segmentation example appears in Fig. 1A.

Manual quality control of raw T1-weighted scans was done following guidelines developed by our group (https://github.com/CoBrALab/documentation/wiki/Motion-Quality-Control-(QC)-Manual).^31,41^ WMHV values were extracted in native space and divided by total brain volume and log-transformed to address skewness, as suggested by previous studies.^42^

### Cardiometabolic data

Cardiometabolic risk factors were selected from the available UK Biobank data to be representative measures of known risk factors for WMHs and dementia.^20,21,43^ We included a total of 17 variables in the categories of alcohol consumption, blood pressure (BP), body size, diabetes, physical activity and smoking. Distributions of variables were visually inspected and, where needed, variables were transformed, binarized, log-transformed or averaged. When necessary, data were recoded so that higher values consistently indicated an overall higher burden. For example, for alcohol intake frequency (field 1558), the initial data was coded on a 6-point scale where less alcohol intake had the highest value. We recoded this variable so that a lower alcohol intake yielded a value of 1 and the highest value possible was 6. We note that data on BP medication is binary, as we did not have access to dosage information. This data could thus include a subset of participants taking low-dose BP medication for headaches or migraines. A full overview of all included cardiometabolic variables, their corresponding UK Biobank field IDs and the performed transformations can be found in Supplementary Table 1. Total household income before tax was used to control for income. The total number of participants that were included per cardiometabolic variable can be found in Supplementary Table 2. When cardiometabolic variables were used as covariates, we imputed missing values using multivariate imputation by chained equations with five repetitions with the *mice* package in R.^44^

### Statistical analyses

All statistical analyses were performed using R (version 4.1.2). Multiple linear mixed effects models with the *lmer* function were used for all statistical tests using z-scored continuous variables to obtain standardized beta coefficients. Analyses included MHT, income (as a proxy for socio-economic status), a random effect of MRI site and second-order polynomial age as covariates (unless stated otherwise).

#### Samples

Age-matched groups were created as in Costantino *et al*. (2023), using the k-nearest-neighbours algorithm from the MatchIt package in R.^45^ Propensity scores based on the generalized linear model were used to estimate the distance between individuals.

The full unmatched sample consists of all individuals who met the inclusion criteria (Table 1A). To create the nearest-neighbour age-matched sample (Table 1B), the PRE group was used as a reference, and the SURG group was matched to this group. The POST group was then matched to the resulting participants using the same method. Figure 1B shows a comparison of the age distributions before and after age-matching, and Fig. 1C shows the WMHV by group distributions. The exact age-matched sample (Table 1C) was created using the same method, but an exact match was required instead of the nearest match, making the age-matching more strict to rule out any artifactual results due to the matching technique. For the POST-SURG age-matched sample (Table 1D), the POST and SURG groups were matched directly. These same age-matched individuals were included when analysing POST and SURG groups separately (Table 1D^x^ and 1D^y^).

Most analyses were done using the aforementioned three datasets. We performed two additional analyses. First, we analytically replicated the study by Lohner and colleagues (2022).^8^ We matched their processing steps by normalizing UKB-derived WMHV values using white matter volume instead of total brain volume, restricting the age range by excluding participants over 59, and not differentiating between natural and surgical menopause (Table 1E). Second, as the loss of oestrogens could theoretically take time to influence vascular brain health, we created a sample by first excluding all participants who had menopause less than 5 years prior to scanning to keep within the bounds of our age-matched dataset (POST: *n* = 1547; SURG: *n* = 68), and then age-matched using the same method as for the nearest-neighbour age-matched sample (Table 1F).

#### Differences in WMHV across menopausal status

First, we investigated differences in WMHV across menopausal status using different analysis schemes. The full unmatched sample was analysed using both linear and second-order polynomial age covariates to investigate common age correction strategies in the literature. The Lohner replication sample was analysed with a linear age covariate, additionally covarying for hypertension, smoking and BMI to match their analysis scheme. All other samples (see Table 1) were analysed using a second-order polynomial age covariate. We carried out additional analyses covarying for all cardiometabolic factors, performing pooled linear regressions (i.e. averaging the coefficients across the five imputed datasets).

#### Effect of age at menopause and MHT use on WMHV

We examined the associations between age at menopause and WMHV in the POST and SURG samples separately, to examine potential effects of age at menopause, as well as the interaction between group and age at menopause in the POST–SURG age-matched sample to explore whether the effect of age at menopause on WMHV is different in natural and surgical menopause. We tested the relationship between MHT and WMHV in the POST and SURG samples separately, as well as the interactions of MHT by group in the POST–SURG age-matched sample. We also examined the interaction of MHT by age at menopause in the POST and SURG samples separately.

#### Effect of menopausal status on the relationships between cardiometabolic factors and WMHV

We investigated the effect of cardiometabolic factors on WMHV across menopausal status. To confirm the previously reported effects of cardiometabolic variables on WMHV in women,^20-22^ we assessed these relationships in the full unmatched sample. Further, we explored cardiometabolic factor by group interactions in the nearest-neighbour age-matched sample to assess whether the suggested protective effect of a healthy cardiometabolic profile depends on the menopausal status. Additionally, we examined whether usage of blood pressure medication has an effect on WMHV and whether this effect was the same in the different menopausal groups (analysis in the nearest-neighbour age-matched sample). A full list of all models can be found in Table 2. Multiple comparisons were corrected using the false discovery rate (FDR) correction^46,47^ in each analysis separately.

## Results

After exclusions, the final sample consisted of 9560 women across menopausal status: 674 in the PRE group, 7992 in the POST group and 894 in the SURG group. A full description of the different samples used is displayed in Table 1, and the linear models used for each analysis and sample are detailed in Table 2.

### Differences in WMHV across menopausal status

**Figure 2:**
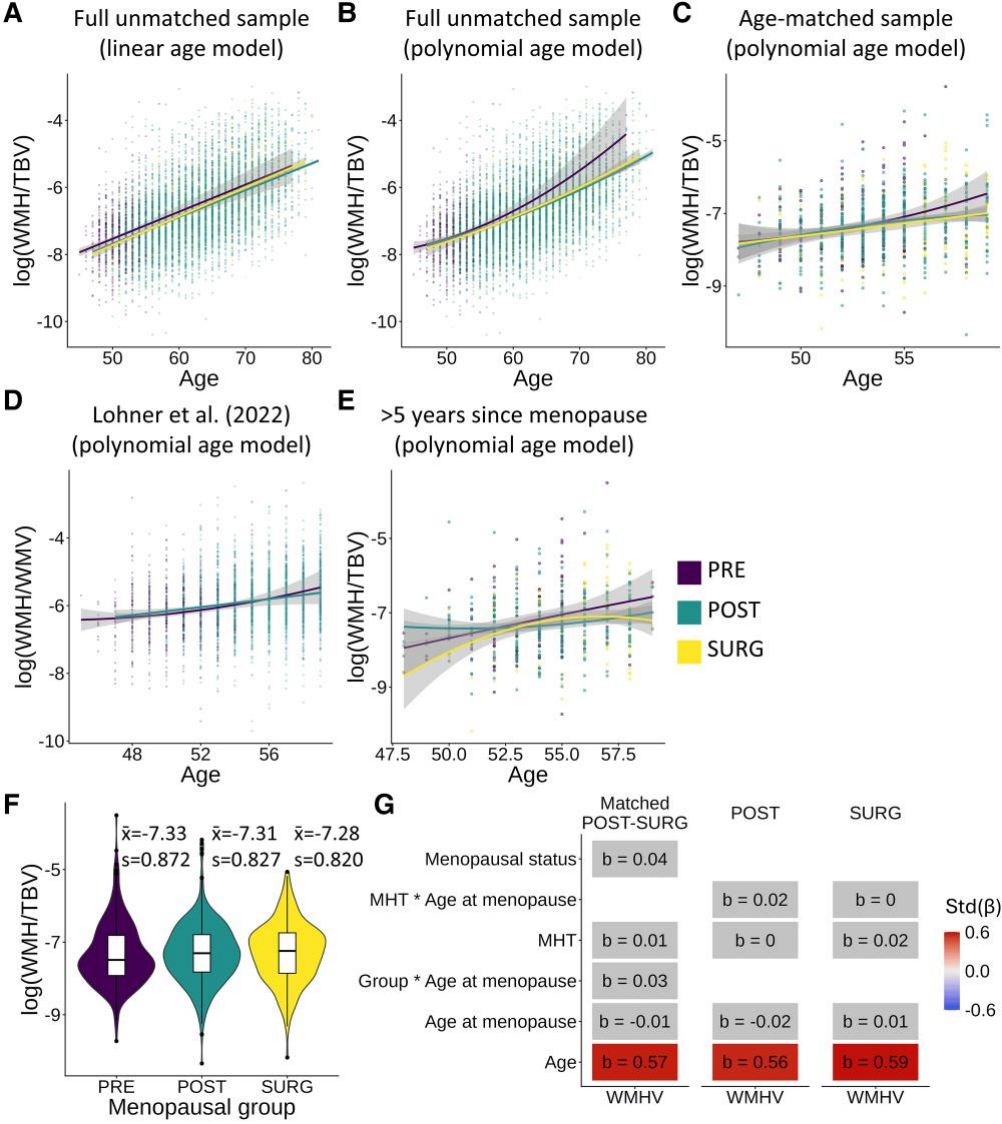
**Age effects of WMHV by menopausal groups and associations with age at menopause and MHT use. (A–E)** Line graphs showing the relationship between age and WMHV, separated by group, for five different models. (**A, B)** Full unmatched sample (*n* = 9560). (**C)** Nearest-neighbour age-matched sample (*n*  *=* 908). (**D)** Lohner *et al*. (2022) replication sample (*n* = 3669). (**E)** >5 years after menopause age-matched sample (*n* = 526). (**F**) Violin plot showing the absence of differences across menopausal groups in WMHV in the age-matched sample (*n* = 908). This is for visualization only, as models run in the main text include covariates. Plots **A–G** are shown for visualization purposes only and do not directly reflect models run with covariates. (**G**) Summary of the results of MHT use and age at menopause in the POST–SURG age-matched sample (*n* = 1788). The color and number in the cell represent the standardized beta from the linear model. Grey cells represent an FDR-corrected *P*-value over 0.05. Income and MRI site were corrected in each model. WMH: White matter hyperintensity. WMHV: White matter hyperintensity volume. TBV: Total brain volume. WMV: White matter volume. MHT: Menopause hormonal therapy. PRE: Premenopausal women. POST: Women who underwent natural menopause. Surg: Women who underwent surgical menopause.

In the full unmatched sample (Table 1A; Fig. 2A), covarying for linear age effects showed evidence for lower WMHV in postmenopausal women relative to premenopausal women (POST: *β* = −0.17, *P* < 0.001; SURG: *β* = −0.15, *P* = 0.001). Adding a quadratic age covariate (Fig. 2B) attenuated these results and rendered them non-significant (POST: *β* = −0.08, *P* = 0.052; SURG: *β* = −0.06, *P* = 0.220), and additionally correcting for cardiometabolic factors resulted in small but significant or trending effects (POST: *β* = −0.09, *P* = 0.036; SURG: *β* = −0.10, *P* = 0.054). Age by group graphs are shown for visualization purposes only in Fig. 2 (these graphs do not reflect linear models fit with covariates). These results are contrary to previous findings reporting higher WMHV after the menopausal transition.^8,25^

In the nearest-neighbour age-matched sample (Table 1B; Fig. 2C; Fig. 2F), results showed similar directionality (i.e. higher WMHV in postmenopausal women) but did not reach significance with (POST: *β* = −0.05, *P* = 0.484; SURG: *β* = −0.15, *P* = 0.116) or without (POST: *β* = −0.06, *P* = 0.436; SURG: *β* = −0.13, *P* = 0.249) cardiometabolic covariates. In the exact age-matched sample (Table 1C), results reached significance in the SURG group only, showing moderate effect sizes with (POST: *β* = −0.08, *P* = 0.385; SURG: *β* = −0.27, *P* = 0.026) and without (POST: *β* = −0.12, *P* = 0.275; SURG: *β* = −0.31, *P* = 0.030) cardiometabolic covariates. When comparing age-matched natural and surgical postmenopausal groups (Table 1D), no significant differences were detected, with (SURG: *β* = 0.02, *P* = 0.634) and without (SURG: *β* = 0.05, *P* = 0.299) cardiometabolic covariates.

In sensitivity analyses, when matching the Lohner *et al*. (2022) methods and using UK Biobank (UKB)-derived WMHV values (Table 1E; Fig. 2D), there were no significant differences between pre- and postmenopausal women (*β* = 0.06, *P* = 0.146). When only including postmenopausal women who had menopause at least 5 years prior to scanning (Table 1F; Fig. 2E), small-to-moderate effects indicating lower WMHV in postmenopausal women were found with (POST: *β* = −0.23, *P* = 0.021; SURG: *β* = −0.39, *P* = 0.003) and without (POST: *β* = −0.25, *P* = 0.028; SURG: *β* = −0.41, *P* = 0.007) cardiometabolic covariates.

In sum, we did not detect that postmenopausal women have higher WMHV than premenopausal women when accounting for age using four different analysis strategies or when using analytical strategies previously used by other groups. We instead find higher WMHV in premenopausal women, especially as compared to surgical menopause.

### Effect of age at menopause and MHT use on WMHV

Associations between MHT, age at menopause and WMHV were analysed in the POST–SURG age-matched sample (Table 1D) and are summarized in Fig. 2G. No associations were found between age at menopause and WMHV across groups (*β* = 0.00, *P* = 0.959) or within the POST (*β* = 0.00, *P* = 0.893) and SURG (*β* = 0.01, *P* = 0.840) groups. Furthermore, no group by age at menopause interaction was found (*β* = 0.01, *P* = 0.735). No main effect was found for MHT across groups (*β* = 0.03, *P* = 0.507), or in the separate POST (*β* = 0.03, *P* = 0.580) and SURG (*β* = 0.02, *P* = 0.805) groups. Furthermore, no MHT by group interaction (*β* = −0.01, *P* = 0.943) or MHT by age at menopause interaction (POST group: *β* = 0.01, *P* = 0.227; SURG group: *β* = 0.00, *P* = 0.786) were found. In sum, we did not detect that age at menopause or use of MHT influenced WMHV, whether within or differently between natural and surgical menopause groups.

### Effect of menopausal status on the relationships between cardiometabolic factors and WMHV

**Figure 3:**
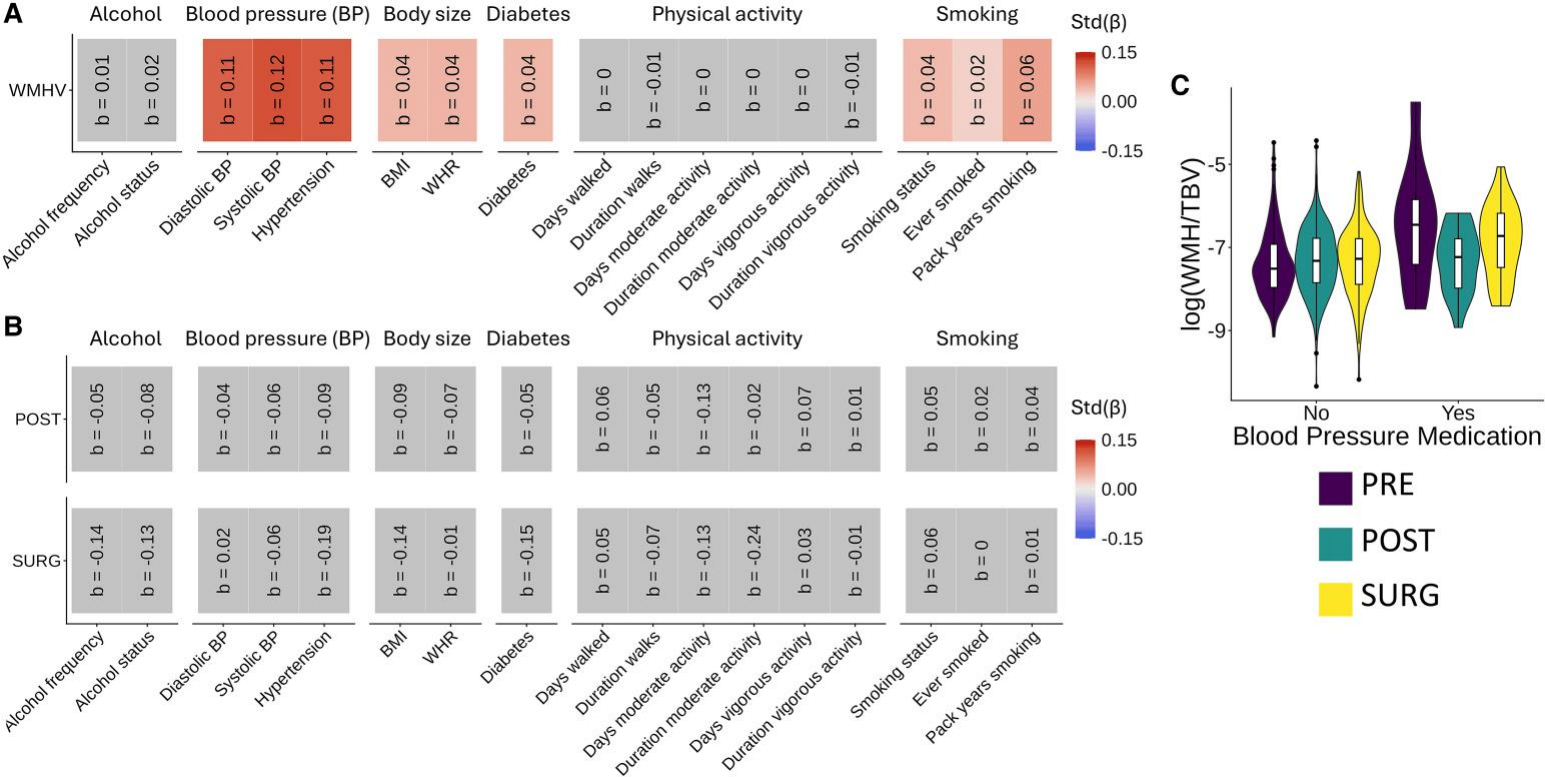
**Effects of menopausal status on the relationships between cardiometabolic factors and WMHV.** The colour and number in each cell represent the standardized beta. Grey cells represent an FDR-corrected *P*-value over 0.05. Each linear model was corrected for income, polynomial age effects, MHT and MRI site. Field IDs and transformations are detailed in Supplementary Table 1. (**A)** Associations between WMHV and cardiometabolic factors in the full unmatched sample (*n* = 9560). (**B)** Interactions between cardiometabolic factors and menopausal groups on WMHV in the nearest-neighbour age-matched sample (*n* = 908). (**C)** Differences in WMHV across groups in users and non-users of blood pressure medication in the nearest-neighbour age-matched sample (*n* = 908). BP: Blood pressure. BMI: body mass index. WHR: Waist–hip ratio. WMH: White matter hyperintensity. TBV: Total brain volume. PRE: Premenopausal women. POST: Women who underwent natural menopause. Surg: Women who underwent surgical menopause.

All cardiometabolic variables tested, except those related to physical activity and alcohol use, were significant predictors of WMHV in the full unmatched sample (Table 1A; Fig. 3A). In the nearest-neighbour age-matched sample (Table 1B), no significant group by cardiometabolic factor interaction was found after FDR correction (Fig. 3B), indicating that we did not detect that the positive influence of a healthy cardiometabolic profile on WMHV differed across menopausal status. However, we found a significant group by blood pressure medication interaction (Fig. 3C), indicating that the effect of blood pressure medication is significantly stronger (i.e. associated with higher WMHV) in premenopausal women relative to naturally postmenopausal women (*β* = −0.32, *P* = 0.002). The interaction did not reach significance for surgically postmenopausal women (*β* = −0.17, *P* = 0.089).

## Discussion

In the present study, we investigated the relationships between WMHV and menopausal status and type, age at menopause, MHT and cardiometabolic factors in a large, well-powered sample obtained from the UK Biobank.

A key takeaway from our findings is the importance of appropriate age correction when studying the menopausal transition. Specifically, using a non-linear age correction significantly altered the interpretation of the results, as it better modelled age-related trends in the unmatched sample. We also observed variations in the results when we performed age-matching procedures. Age-matching is a crucial aspect of studying the menopausal transition, since age is simultaneously the cause of menopause and large changes in brain morphology,^48^ thus making it an important confounder of studies linking menopause to the brain. Robust age corrections are crucial, as any residual age variance may lead to incorrect conclusions. This might explain our diverging results relative to another study using UK Biobank data, which found higher WMHV in postmenopausal women but did not age-match.^25^

Intriguingly, our study finds either no significant differences between menopausal groups (in the nearest-neighbour age-matched sample and the Lohner *et al*. (2022)^8^ replication sample) or we find evidence of higher WMHV in premenopausal women, especially as compared to surgically postmenopausal women (in the unmatched sample, the exact age-matched sample, and the sample with postmenopausal women >5 years after menopause). These results do not align with previously reported effects of higher WMHV in postmenopausal women.^8,25^ We hypothesize that menopause may have inverted U-shaped non-linear effects on brain health, with an initial increase in WMHV in the early stages of menopause, which gets resolved as the brain adapts to the hormonal changes (e.g. decreases in oestrogen levels), as one study demonstrated.^35^ Shrinking of WMHs has been consistently reported in previous studies.^49^ Furthermore, a large proportion of women classified as premenopausal may in fact be currently undergoing menopause, as the UK Biobank contains no data on perimenopause. Thus, most ‘premenopausal’ women who underwent brain MRI in the UK Biobank (age range: 45–77) may have been perimenopausal and in a critical period of increased brain vulnerability due to fluctuations in hormone levels (i.e. at the peak of the non-linear effect), which could be a specific idiosyncracy of the UK Biobank menopausal data and may explain the reported negative results linking brain markers to menopausal status in that dataset, especially when performing age-matching.^31,50^ This could also explain why effects are higher relative to the surgical menopause group, who have, on average, a longer time since menopause (8.34 years in the exact age-matched sample) relative to naturally postmenopausal women (3.63 years), and in the group excluding women who underwent menopause less than 5 years before scanning. These women may be past the vulnerable brain state, as they have had more time to adapt to the hormonal changes following menopause and the loss of ovarian function and reduction in ovarian hormones (i.e. they are at the later end of the non-linear effect). It is unlikely that these effects in the SURG group are driven by MHT use, as we find no main effect of MHT and we covary for MHT use in the analysis comparing WMHV across groups. This hypothesis should be tested in future studies that are adequately designed and powered to specifically isolate the impact of all stages of the menopause transition on biological phenotypes.

We then investigated the effect of age at menopause and MHT use on WMHV in naturally and surgically menopausal women. Contrary to previous studies, no effect of age at menopause was found in any group.^23,24^ However, these previous studies had smaller samples,^24^ and used a categorical instead of a continuous measure for age at menopause.^23,24^ Furthermore, we found no effect of MHT on WMHV, which is consistent with other literature that has reported mixed or negative findings on the protective effects of MHT on brain health.^23,33,34^ However, due to the absence of data regarding type, dosage and duration of MHT in the UK Biobank, we cannot exclude a more subtle, dose-dependent effect of MHT use on WMHV,^32,51^ and further research is needed to gain a more comprehensive understanding of its effects.

Our findings support the reported relationships between cardiometabolic factors and WMHV. Specifically, the risk factors linked to WMHV in the present study were in the categories of blood pressure, body size, diabetes and smoking. Interestingly, contrary to previous reports,^52^ no effects were observed for physical activity. Notably, Livingston *et al*. (2020) reported that physical inactivity was a risk factor for dementia.^43^ However, Habes and colleagues (2016) also reported no significant interactions between physical activity and WMHV.^20^ This suggests that while physical activity may be a risk factor for general brain aging and dementia, there may not be an association with WMHV specifically. However, another possible explanation could be that the physical activity data in the UK Biobank reflects the current level of activity, which may not accurately represent the physical activity patterns throughout life. A measure of activity throughout the lifespan may be more suitable for drawing conclusions. We observed that the effect of blood pressure medication is associated with higher WMHV to a larger extent in premenopausal women relative to postmenopausal women. This could indicate that very high blood pressure that requires medication may particularly impact WMHV in premenopausal (or perimenopausal) women, who may be in a critical period of brain vulnerability due to a lack of cerebral blood flow. However, generally, we provide evidence that the protective effects of a healthy cardiometabolic profile on brain health, using WMHV as a proxy, do not change after the menopausal transition. Taken together, our results suggest that the critical factors for cerebrovascular health in aging women are factors other than the menopausal transition, such as cardiometabolic risk factors.

The present study should be considered in light of its limitations. First, the UK Biobank contains no data on perimenopause, which may have led to mislabelling of individuals in the premenopausal group and may underlie the unexpected findings of higher WMHV in premenopausal women. Given that perimenopause typically lasts around 4 years^53^ and is associated with fluctuations of sex hormone levels,^54^ the inclusion of perimenopausal individuals within the premenopausal group could have biased the groupings. No conclusions can be made on perimenopausal women based on these findings. Furthermore, data on menopause and age at menopause relied on self-report, raising uncertainties about the reliability of this data, and no data on menopause-related symptoms (e.g. hot flashes) were available. For instance, older individuals who experienced menopause decades ago may not be able to accurately recall their precise age at menopause. However, most of these individuals will not have been included in our younger, age-matched sample. Lastly, the UK Biobank sample is not gender diverse (i.e. no transgender or intersex individuals are explicitly included and no self-identification questionnaires regarding gender were used) and consists predominantly of self-reported as ‘White British people’, thus our findings may not be generalizable to a broad, diverse population in terms of gender, culture, nationality or ethnicity.

In summary, our study contributes important knowledge to the field and to women-specific health factors in aging, highlighting the interplay between menopausal status, brain health and cardiometabolic risk factors. Research has historically overlooked women’s health, and this study adds to the body of literature that aims to fill this critical gap. Furthermore, our study underscores the importance of methodological considerations in future research, reveals idiosyncrasies in the UK Biobank menopausal data, and emphasizes the importance of a healthy cardiometabolic profile throughout life for maintaining a healthy brain, specifically in women’s health.

## Supplementary Material

fcaf475_Supplementary_Data

## Data Availability

Data were obtained from the UK Biobank, which is openly available to researchers following registration and approval through the UK Biobank Access Management System (https://www.ukbiobank.ac.uk/enable-your-research/register). All pipelines used for processing the MRI data are publicly available tools, referenced throughout the manuscript. We share all code used to run analyses, as well as raw results from linear models and raw visualizations, on GitHub (https://github.com/CoBrALab/WMH_UKB_menopause).
