# Supplementary material for: Investigating links between white matter hyperintensities and menopausal status using robust age-correction methods in UK Biobank
Source: Brain Commun. 2025 Dec 5;7(6):fcaf475. doi: 10.1093/braincomms/fcaf475 (PMC12715771; doi:10.1093/braincomms/fcaf475)
Supplement: fcaf475_Supplementary_Data [file fcaf475_supplementary_data.docx]

**Supplementary Materials**

### Supplementary Methods 1 - MRI acquisition parameters

MRI acquisition parameters for UK Biobank (same parameters across the three sites)

1. **T1-weighted**: Sagittal 3D MPRAGE; in-plane acceleration factor (R) = 2; inversion time (TI) = 880 ms; repetition time (TR) = 2000 ms; resolution = 1 x 1 x 1 mm
2. **T2-weighted Fluid-attenuated inversion recovery (FLAIR)**: Sagittal 3D SPACE; R = 2; partial Fourier (PF) = 7/8; fat saturation; TI = 1800 ms; TR = 5000 ms; elliptical k-space scanning; resolution = 1.05 x 1 x 1 mm
3. **Diffusion-weighted imaging (DWI)**: SE-EPI; multiband factor (MB) = 3; R = 1; TE = 92 ms; TR = 3600 ms; PF = 6/8; fat saturation; b-values: 5 x b = 0 s/mm2, 50 x b = 1000 s/mm2, 50 x b = 2000 s/mm2 (100 distinct diffusion directions); phase-encoding reversed data acquired; resolution = 2 x 2 x 2 mm

**Supplementary Methods 2 - Image processing**

First, both the raw T1w and FLAIR images were denoised[^1^](https://paperpile.com/c/sXEpUh/IqSVT), field-inhomogeneity corrected[^2^](https://paperpile.com/c/sXEpUh/u5VNr), and intensity normalized between 0 and 100. A brain mask was computed with the BEaST algorithm[^3^](https://paperpile.com/c/sXEpUh/gzXeM). The FLAIR images were rigidly registered to the T1w images with the Advanced Normalization Tools (ANTs) pipeline[^4^](https://paperpile.com/c/sXEpUh/aBqUm). Second, to ensure high registration accuracy to a common template space, we generated a custom UK Biobank template with the T1w images of 100 males and 100 females with representative age distributions[^5^](https://paperpile.com/c/sXEpUh/dSUYC). Third, to enhance the registration accuracy in the white matter tracts, which has low contrast on T1w images, we leveraged the fractional anisotropy (FA) maps derived from diffusion-weighted imaging[^6^](https://paperpile.com/c/sXEpUh/uP7WH), which were further denoised, super-sampled to 1 mm isotropic in resolution[^7^](https://paperpile.com/c/sXEpUh/GL2DU), and rigidly registered to the T1w image[^4^](https://paperpile.com/c/sXEpUh/aBqUm). We then performed multispectral non-linear registration, using processed T1w and FA images as inputs, to register to the common template. Fourth, we manually labeled the WMHs of 60 UK Biobank participants, which we then used to retrain the validated Brain Tissue Segmentation (BISON) pipeline[^8^](https://paperpile.com/c/sXEpUh/5kDRh). Applied to the whole cohort, this segmented the brain into 9 tissue types including WMHs using both preprocessed T1w and FLAIR images as inputs (<https://github.com/VANDAlab/BISON-WMH>).

### Supplementary Figure 1

###
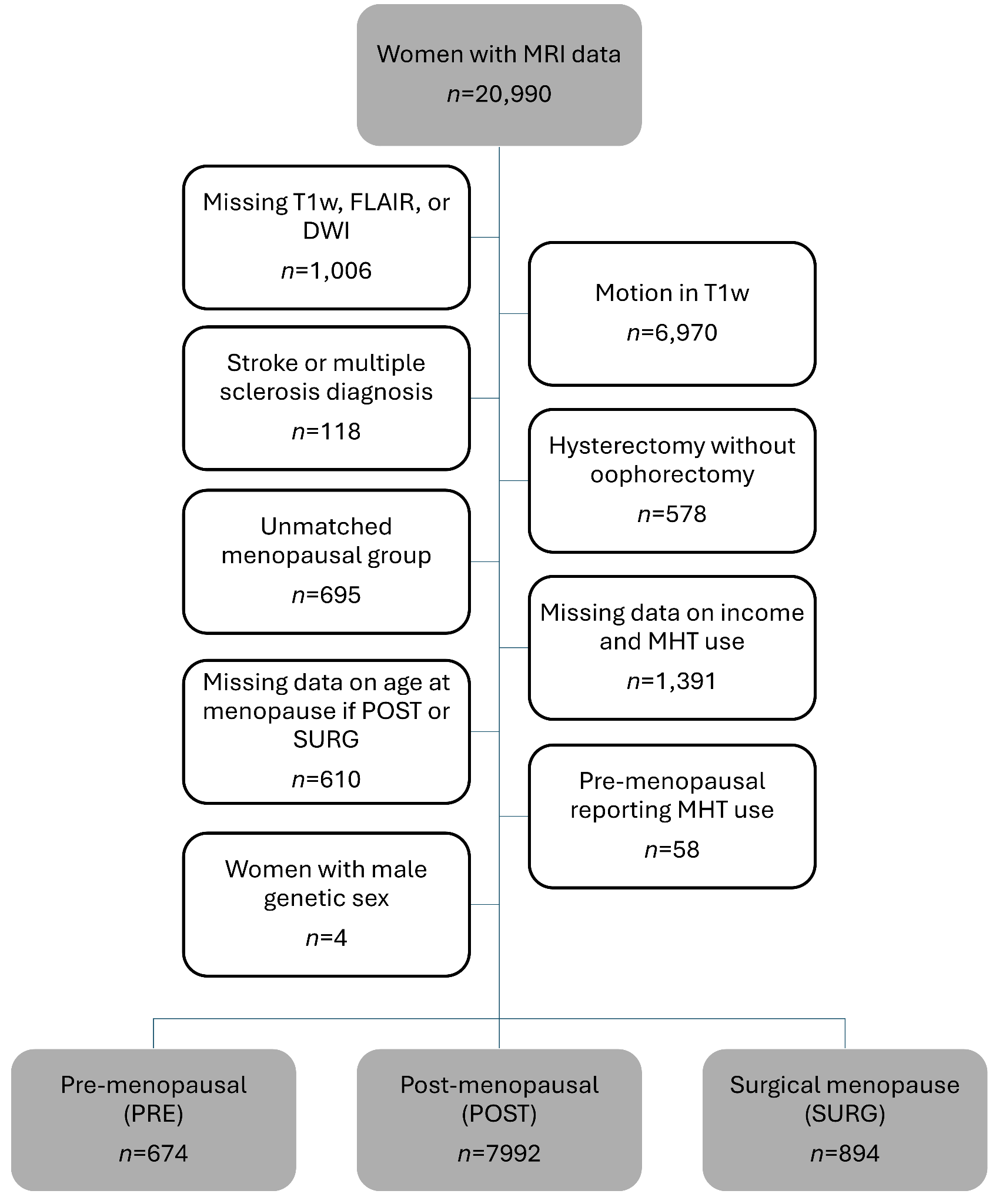


*Flowchart of exclusions.* At each exclusion step, the number of individuals excluded is shown. Exclusion numbers are additive. The original and final sample sizes are shown.

###

| Supplementary Table 1 *Risk factor variables.* | | | | |
| --- | --- | --- | --- | --- |
| Category | Name | Field ID | Variable name | Transformation |
| Alcohol | Alcohol drinker status | [20117](https://biobank.ndph.ox.ac.uk/showcase/field.cgi?id=20117) | alcohol_status | Binarized: never and previous combined into no |
|  | Alcohol intake frequency | [1558](https://biobank.ndph.ox.ac.uk/showcase/field.cgi?id=1558) | alcohol_frequency | - |
| Blood pressure | Regularly takes blood pressure medication | [6153](https://biobank.ndph.ox.ac.uk/showcase/field.cgi?id=6153) | BP_medication | Binarized to yes and no for blood pressure medication |
|  | Diastolic blood pressure | - | BP_diastolic | Calculated by averaging data fields [94](https://biobank.ndph.ox.ac.uk/showcase/field.cgi?id=94) and [4079](https://biobank.ndph.ox.ac.uk/showcase/field.cgi?id=4079) |
|  | Systolic blood pressure | - | BP_systolic | Calculated by averaging data fields [93](https://biobank.ndph.ox.ac.uk/showcase/field.cgi?id=93) and [4080](https://biobank.ndph.ox.ac.uk/showcase/field.cgi?id=4080) |
|  | High blood pressure diagnosis | 2966 | High_BP | Binarized to yes or no from Age at diagnosis |
| Diabetes | Diabetes diagnosed by doctor | Category 1712 | diabetes | Diabetes ICD-10 code before the MRI visit |
| Obesity | Body mass index (BMI) | [23104](https://biobank.ndph.ox.ac.uk/showcase/field.cgi?id=23104) | BMI | - |
|  | Waist to hip ratio (WHR) | - | WHR | Calculated using waist circumference (data field [48](https://biobank.ndph.ox.ac.uk/showcase/field.cgi?id=48)) and hip circumference (data field [49](https://biobank.ndph.ox.ac.uk/showcase/field.cgi?id=49)) |
| Physical activity | Number of days/week walked 10+ minutes | [864](https://biobank.ndph.ox.ac.uk/showcase/field.cgi?id=864) | days_walked | - |
|  | Duration of walks | [874](https://biobank.ndph.ox.ac.uk/showcase/field.cgi?id=874) | duration_walks | Collapsed at 3.5 SD above mean, log-transformed |
|  | Number of days/week of moderate physical activity 10+ minutes | [884](https://biobank.ndph.ox.ac.uk/showcase/field.cgi?id=884) | days_moderate_activity | - |
|  | Duration of moderate activity | [894](https://biobank.ndph.ox.ac.uk/showcase/field.cgi?id=894) | duration_moderate_activity | Collapsed at 3.5 SD above mean, log-transformed |
|  | Number of days/week of vigorous physical activity 10+ minutes | [904](https://biobank.ndph.ox.ac.uk/showcase/field.cgi?id=904) | days_vigorous_activity | - |
|  | Duration of vigorous activity | [914](https://biobank.ndph.ox.ac.uk/showcase/field.cgi?id=914) | duration_vigorous_activity | Collapsed at 3.5 SD above mean, log-transformed |
| Smoking | Smoking status | [20116](https://biobank.ndph.ox.ac.uk/showcase/field.cgi?id=20116) | smoking_status | Binarized into yes and no |
|  | Past tobacco smoking | [1249](https://biobank.ndph.ox.ac.uk/showcase/field.cgi?id=1249) | past_tobacco | - |
|  | Pack years | [20161](https://biobank.ndph.ox.ac.uk/showcase/field.cgi?id=20161) | pack_years | Never smokers set to zero using data field [20160](https://biobank.ndph.ox.ac.uk/showcase/field.cgi?id=20161), log-transformed |

*Note.* Table shows the categories of the variables, the name and field ID of the variables in the UK Biobank data, the variable name as used in the present study and as seen in Figure 3A-B, and transformations that were performed to obtain the final variables.

| **Supplementary Table 2** *Number of participants included per variable.* | | | | | |
| --- | --- | --- | --- | --- | --- |
| **Variable** | **Full unmatched sample** | **Nearest neighbour age-matched sample** | | | |
|  |  | **Total** | **PRE** | **POST** | **SURG** |
| Age | 9,560 | 908 | 223 | 454 | 231 |
| Income | 9,560 | 908 | 223 | 454 | 231 |
| MHT | 9,560 | 908 | 223 | 454 | 231 |
| WMHV | 9,560 | 908 | 223 | 454 | 231 |
| Age at Menopause | 8,886 | 685 | 0 | 454 | 231 |
| Time Since Menopause | 8,886 | 685 | 0 | 454 | 231 |
| Days Walked | 9,527 | 905 | 221 | 453 | 231 |
| Duration of Walks | 8,769 | 831 | 203 | 419 | 209 |
| Days Moderate Activity | 9,390 | 894 | 219 | 447 | 228 |
| Duration Moderate Activity | 8,258 | 761 | 181 | 386 | 194 |
| Days Vigorous Activity | 9,403 | 900 | 222 | 450 | 228 |
| Duration Vigorous Activity | 6,176 | 564 | 142 | 291 | 131 |
| Smoking Status | 9,560 | 908 | 223 | 454 | 231 |
| Past Tobacco Use | 9,390 | 886 | 219 | 448 | 219 |
| Pack Years | 6,675 | 624 | 151 | 306 | 167 |
| Alcohol Status | 9,560 | 908 | 223 | 454 | 231 |
| Alcohol Frequency | 9,559 | 908 | 223 | 454 | 231 |
| Diabetes | 9,560 | 908 | 223 | 454 | 231 |
| BMI | 9,055 | 853 | 211 | 428 | 214 |
| BP Diastolic | 7,335 | 690 | 170 | 349 | 171 |
| BP Systolic | 7,335 | 690 | 170 | 349 | 171 |
| BP Medication | 9,560 | 908 | 223 | 454 | 231 |
| Hypertension | 9,560 | 908 | 223 | 454 | 231 |
| WHR | 9,266 | 883 | 216 | 442 | 225 |

### Supplementary References

1. [Manjón JV, Coupé P, Martí-Bonmatí L, Collins DL, Robles M. Adaptive non-local means denoising of MR images with spatially varying noise levels. *J Magn Reson Imaging*. 2010;31(1):192-203.](http://paperpile.com/b/sXEpUh/IqSVT)

2. [Sled JG, Zijdenbos AP, Evans AC. A nonparametric method for automatic correction of intensity nonuniformity in MRI data. *IEEE Trans Med Imaging*. 1998;17(1):87-97.](http://paperpile.com/b/sXEpUh/u5VNr)

3. [Eskildsen SF, Coupé P, Fonov V, et al. BEaST: Brain extraction based on nonlocal segmentation technique. *Neuroimage*. 2012;59(3):2362-2373.](http://paperpile.com/b/sXEpUh/gzXeM)

4. [Avants BB, Tustison NJ, Song G, Cook PA, Klein A, Gee JC. A reproducible evaluation of ANTs similarity metric performance in brain image registration. *Neuroimage*. 2011;54(3):2033-2044.](http://paperpile.com/b/sXEpUh/aBqUm)

5. [Fonov VS, Evans AC, McKinstry RC, Almli CR, Collins DL. Unbiased nonlinear average age-appropriate brain templates from birth to adulthood. *Neuroimage*. 2009;47:S102.](http://paperpile.com/b/sXEpUh/dSUYC)

6. [Alfaro-Almagro F, Jenkinson M, Bangerter NK, et al. Image processing and Quality Control for the first 10,000 brain imaging datasets from UK Biobank. *Neuroimage*. 2018;166:400-424.](http://paperpile.com/b/sXEpUh/uP7WH)

7. [Manjón JV, Coupé P, Buades A, Fonov V, Louis Collins D, Robles M. Non-local MRI upsampling. *Med Image Anal*. 2010;14(6):784-792.](http://paperpile.com/b/sXEpUh/GL2DU)

8. [Dadar M, Collins DL. BISON: Brain tissue segmentation pipeline using T1 -weighted magnetic resonance images and a random forest classifier. *Magn Reson Med*. 2021;85(4):1881-1894.](http://paperpile.com/b/sXEpUh/5kDRh)
